# Errors in Figure 2 and Supplement 1

**DOI:** 10.1001/jamanetworkopen.2026.12307

**Published:** 2026-04-17

In the Original Investigation titled “Global Burden of Violence Against Transgender and Gender-Diverse Adults: A Systematic Review and Meta-Analysis,”^1^ published January 21, 2026, there were errors in Figure 2 and Supplement 1. In Figure 2, panel A should have been labeled “Ever experienced physical or sexual violence” and panel B should have been labeled “Recently experienced physical or sexual violence.” Supplement 1 was missing the eFigure titled, “Meta-Analyses of Physical Violence Against Transgender and Gender-Diverse Adults,” which includes a panel showing data for transgender and gender-diverse adults who have ever experienced physical violence and another panel showing data for those who have recently experienced physical violence. This article has been corrected.^1^
